# Transcriptome analysis of rice resistant and susceptible near-isogenic lines in response to infection by *Xanthomonas oryzae* pv. *oryzicola*

**DOI:** 10.3389/fpls.2025.1610315

**Published:** 2025-09-18

**Authors:** Hui Zhang, Min Tang, Yao Wan, Ziqiu Deng, Xuemei Qin, Jieyi Huang, Xuan Wei, Rongbai Li, Fang Liu

**Affiliations:** State Key Laboratory for Conservation and Utilization of Subtropical Agro-Bioresources, College of Agriculture, Guangxi University, Nanning, China

**Keywords:** rice, bacterial leaf streak (BLS), transcriptome sequencing, differentially expressed genes (DEGs), near-isogenic line

## Abstract

Rice bacterial leaf streak (BLS), caused by gram-negative bacterium *Xanthomonas oryzae* pv. *oryzicola* (*Xoc*), is one of the most destructive quarantine diseases internationally. Effectively utilizing BLS resistance genes from wild rice to breed resistant varieties can solve the problem of controlling BLS at its source. In this study, resistant near-isogenic line NIL-*bls2* (abbreviated as R) and susceptible near-isogenic line NIL-*BLS2* (abbreviated as S) in BC_4_F_3_ were constructed by using Guangxi common wild rice material DY19, which carries the BLS resistance gene *bls2* and susceptible indica rice variety 9311. Transcriptome sequencing was used to analyze the molecular interaction mechanism of R and S in response to infection by a highly pathogenic *Xoc* strain gx01. The results showed that between R and S, there were 218 differentially expressed genes (DEGs) at 12 hours post inoculation (hpi), 170 DEGs at 24 hpi, and 329 DEGs at 48 hpi after inoculation. GO and KEGG enrichment analysis revealed that the following changes occurred in R compared to S after *Xoc* infection: At 12 hpi, R enhanced cell wall toughness by synthesizing lignin; increased the ability to recognize and bind bacterial flagellin flg22, activating multiple immune responses of downstream signal transmission; and promoted wound healing by enhancing the synthesis of traumatic acid. At 24 hpi, R synthesized a large number of diterpenoid phytoalexins, up-regulated genes related to disease resistance protein PR1 and heat shock protein HSP90B, and activated jasmonic acid and salicylic acid-dependent signal transduction pathways. At 48 hpi, R carried out a defense reaction by strengthening the cell wall, enhancing jasmonic acid synthesis, synthesizing monoterpenes and isoquinoline alkaloids, etc. Taken together, *bls2* was proposed to regulate both PTI- and ETI-related genes through multi-level defense system, including plant hormone-mediated regulation, antimicrobial phytoalexin biosynthesis, and structural barrier reinforcement, to against *Xoc* infection. It laid a theoretical foundation for further in-depth research into the molecular mechanism of the BLS resistance gene *bls2*.

## 1 Introduction

Bacterial leaf streak (BLS) caused by *Xanthomonas oryzae* pv. *oryzicola* (*Xoc*) is the fourth major rice disease after rice blast, bacterial blight and sheath blight. It has the characteristics of early onset in the growth cycle, rapid spread, strong destructiveness and frequent recurrence. In severely affected areas, BLS can lead to a 40% - 60% reduction in rice yield (Niño-Liu et al., 2006).

*Xoc* is a pathogenic variant of the genus *Xanthomonas* in the family *Xanthomonadaceae*. It is a gram-negative, aerobic, and rod-shaped bacterium without a capsule or spores. It moves using its flagella and invades rice mainly through leaf wounds or stomata. Compared with the use of microbicides and other methods, the use of disease-resistance genes to cultivate disease-resistant varieties is the most effective, economical and environmentally friendly method to prevent disease outbreaks (Zhang, 2007). At present, most BLS resistance genes identified from cultivated rice (*Oryza sativa*) are quantitative trait loci (QTLs). However, their relatively minor phenotypic effects have posed significant challenges for both gene cloning and subsequent genetic applications. There are only six major resistance genes of rice BLS reported in the literature, including two dominant genes (*Xo1*, *Xo2*) and three recessive genes (*bls1*, *bls2, qBlar5a*), and one non-host resistance gene *Rxo1* (Read et al., 2020; Shi et al., 2019; Chen et al., 2022; Ma et al., 2021; Xie et al., 2014; Zhao et al., 2005). In our previous study, a rice accession (DY19), which were derived from Guangxi common wild rice (*Oryza rufipogon* Griff.), was screened and identified to be highly resistant to BLS. It was shown that the BLS resistance in this material was controlled by a major gene *bls2* (Shi et al., 2019). The major resistance genes represent an important type of resistance resource that is more amenable to breeding utilization. Guangxi is one of the provinces with the largest number of wild rice distribution in China, and wild rice resources are abundant (Deng et al., 2012). Common wild rice has strong resistance and a wide range of resistance spectrum. Currently, excellent resistance sources against major diseases and pests prevalent in cultivated rice can be identified in common wild rice (Li and Qin, 1994). Since common wild rice shares the same AA genome with cultivated rice, superior resistance genes *bls2* identified in common wild rice can be readily transferred into cultivated rice through hybrid breeding approaches. Research on this gene will provide both genetic resources and theoretical foundations for breeding rice varieties resistant to BLS.

Plants have evolved two innate immune defense mechanisms in response to pathogens invasion: broad-spectrum immune defense mechanisms and specific immune defense mechanisms, known as pathogen-associated molecular pattern (PAMP)-triggered immunity (PTI) and effector-triggered immunity (ETI). Receptor-like proteins (RLPs) and receptor-like kinases (RLKs) on the surface of plant cells can directly sense PAMPs, such as bacterial flagellin (Boutrot and Zipfel, 2017), to initiate PTI (Sun et al., 2013). PTI include: the burst of reactive oxygen species (ROS), an increase in intracellular calcium concentration, activation of mitogen-activated protein kinase (MAPK), synthesis of phytoalexin antibiotic compound, pathogenesis-related (PR) gene induction, transcriptional regulation, stomatal immunity, enhanced cell wall toughness by enhancing callose deposition and/or lignin accumulation (Kishi-Kaboshi et al., 2018; Staskawicz et al., 1995). ETI is controlled by plant resistance proteins, which directly or indirectly recognize pathogen effectors, such as transcription activator-like effectors (TALEs) (Chisholm et al., 2006; Jones and Dangl, 2006). ETI is rapid and highly resistant, which usually triggers the hypersensitive response (HR). ETI is also associated with other defense responses include the accumulation of toxic metabolites or proteins, changes in hormone levels, etc (Staskawicz et al., 1995). To better defend against the invasion of pathogens, plant PTI and ETI often occur simultaneously, triggering changes in various signaling pathways, leading to the synthesis of antibacterial secondary metabolites such as lignin (Lam et al., 2022; Rosado et al., 2021), chalcone (Han et al., 2017; Lam et al., 2022), phytoalexin (Zi et al., 2014), alkaloids (Cibils Stewart et al., 2022), and defense-related responses such as protein ubiquitination (Smith et al., 2014).

The defense mechanisms of BLS resistance gene against *Xoc* have been extensively investigated. *Xo1*, which encodes a protein containing the nucleotide binding site–leucine rich repeat (NBS-LRR) domain and activates the rice defense response by recognizing TALEs secreted by *Xoc*. However, the resistance mediated by *Xo1* can be suppressed by iTALE (Read et al., 2020). The *bls1* encodes a mitogen-activated protein kinase (OsMAPK6). Overexpression of *BLS1* and low expression of *bls1* showed increase of salicylic acid (SA) and induced expression of defense-related genes, simultaneously increasing broad-spectrum resistance. Moreover, low expression of *bls1* showed increase of jasmonic acid (JA) and abscisic acid (ABA), in company with an increased resistance to *Xoc* strain JZ-8 (Ma et al., 2021). *qBlsr5a* was found to be allelic to the bacterial blight resistance gene *xa5*, which encodes the gamma chain of transcription initiation factor IIA (TFIIAγ). The nucleotide substitutions resulted in a change of the 39^th^ amino acid from valine in the susceptible parent to glutamic acid in the resistant parent (Xie et al., 2014). Some defense-responsive (DR) gene, such as *OsWRKY45–1* and *OsWRKY45-2* (Tao et al., 2009), the polygalacturonase inhibiting protein genes *OsPGIP1* and *OsPGIP4* (Wu et al., 2019; Feng et al., 2016) have been reported to be involved in BLS resistance. The sulfate transporter gene *OsSULTR3;6* (Xu et al., 2021), and the salicylic acid (SA) metabolic enzyme gene *OsF3H03g* and *OsF3H04g* (Wu et al., 2022; 2021) serves as the susceptibility gene when induced by the TALE of *Xoc*. Editing the effector-binding elements (EBEs) in the promoter regions of these susceptibility genes via CRISPR/Cas9 technology can enhance BLS resistance.

RNA-seq technology is used to study the genome-wide transcriptional map at the whole transcriptome level, which can reveal the gene network of trait regulation, and provide data support for the study of the molecular mechanism of disease-resistant genes, thereby offering a theoretical basis for the cultivation of new rice varieties resistant to disease (Jiang et al., 2017; Deng et al., 2018; Formentin et al., 2018; Yang et al., 2020). The defense system of rice against *Xoc* infection is complex, and the research on the molecular mechanism of rice resistance to BLS is at the initial stage, which needs to be further explored. Near-isogenic lines refer to a group of lines with the same or similar background but different traits or genetic bases. Due to the similar genetic background between near-isogenic lines (NILs), the difference in individual phenotypic traits is only caused by the difference of individual chromosome segments or gene loci. Therefore, the construction of NILs is a common means to study gene function and gene-genetic interaction. In this study, transcriptome sequencing was used to analyze the related metabolic pathways and molecular regulatory networks of resistant and susceptible NILs in response to pathogenic *Xoc* strains, so as to provide a theoretical basis for understanding the interaction mechanism between rice and *Xoc*.

## 2 Methods

### 2.1 Experimental materials

In this experiment, a susceptible variety 9311 and a Guangxi common wild rice accession DY19 carrying the BLS resistance gene *bls2* were used as materials. The bacterial strain was the Guangxi *Xoc* strain gx01 with high pathogenicity, which was provided by professor He Yongqiang of the State Key Laboratory for Conservation and Utilization of Subtropical Agricultural Biological Resources. The experiment was carried out in the scientific research base of the College of Agriculture, Guangxi University, adopting the method of single-plant transplanting.

### 2.2 Construction of BLS resistant near-isogenic lines

Guangxi common wild rice material DY19 carrying *bls2* resistance gene was used as the donor parent, and susceptible indica rice varieties 9311 was used as the recipient parent. The progeny population from the cross were inoculated by pricking with Guangxi prevailing *Xoc* strain gx01 at the tillering stage. Then, the high-resistant individuals were screened and backcrossed with 9311, combined with SL03 marker-assisted multi-generation selection (Shi et al., 2019). In BC_4_F_2_ populations, resistance inoculation assays and molecular marker detection were conducted. The plants were resistant to BLS and exhibited the donor parent’s homozygous genotype at molecular marker SL03 were selected for self pollination. Similarly, the plants were susceptible to BLS and carried the recipient parent’s homozygous genotype at SL03 were also selected for self pollination. Subsequently, the BLS resistant and susceptible NILs were obtained in BC_4_F_3_ populations. Finally, the genetic background of the near-isogenic lines was detected by using polymorphic primers evenly distributed on 12 chromosomes of rice. In order to fine map the introgression fragment of the resistant near-isogenic lines, simple sequence repeat (SSR) and insertion–deletion (InDel) markers were designed using the Gramene website (http://www.gramene.org/) and the software Primer 5. The molecular markers analysis follows the method of Shi et al. (2019), with products analyzed by polyacrylamide denaturing gel electrophoresis and visualized by silver staining.

### 2.3 Bacterial inoculation and lesions identification

The methods of bacterial inoculation and lesions identification referenced from Tang et al. (2022). Three rice plants were inoculated with strain gx01, while three rice plants were inoculated with sterile water and used as blank control. Five leaves with the best growth from each plant were selected for inoculation. Among them, 3 leaves were inoculated 3 times per leaf, with a pinhole spacing of 1 cm for the transcription sequencing experiment; the remaining 2 leaves were inoculated once per leaf to measure the length of the lesions. Leaves used in the transcriptome experiment were harvested at 12 hpi, 24 hpi and 48 hpi. Three biological replicates of leaf samples, each containing a pool of three individual plants, were collected for each time points. The lesions were investigated 15 days after inoculation. According to the identification criteria of the International Rice Research Institute (IRRI), the resistance level was identified according to the lesion length (**Table 1**).

**Table 1:** Identification of rice bacterial leaf streak resistance grades.

Lesion length (cm)	Disease index	Resistant type
0	0	Immune (I)
0.1-0.5	1	Highly resistant (HR)
0.6-1.0	3	Resistant (R)
1.0-1.5	5	Mediately resistant (MR)
1.6-2.5	7	Susceptible (S)
>2.5	9	Highly susceptible (HS)

### 2.4 RNA extraction method

The total RNA of the leaves was extracted using the TIANGEN RNA prep Pure polysaccharide polyphenol plant total RNA extraction kit (DP441; TianGen; China). The RNA concentration was measured by Thermo Qubit 3.0 fluorescence quantitative analyzer (Thermo Fisher Scientific, USA). The RIN value of RNA was assessed by Agilent 4200 Tape Station Bioanalyzer (Agilent Technologies, USA) to evaluate its integrity. The purity of RNA was detected by Thermo Scientific NanoDrop spectrophotometer ND8000 (Thermo Fisher Scientific, USA). Qualification criteria: total RNA greater than 1μg, RIN value greater than 7, OD260/OD280 between 1.8-2.1, OD230/OD260 between 0.4-0.5.

### 2.5 RNA library preparation and sequencing

The RNA libraries were prepared using the VAHTS Universal V6 RNA-seq Library Prep Kit for Illumina (NR604; Vazyme Biotech Co., Ltd; China) following the manufacturer’s instructions. After library construction, fragment size and concentration were assessed using an Agilent 4200 Tape Station Bioanalyzer (Agilent Technologies, USA) and ABI Quant Studio 6 Flex Real-Time PCR (Thermo Fisher Scientific, USA) for quality control. Qualification criteria: library fragment size between 300–500 bp, molar concentration greater than 10 nM. Once the libraries passed quality control, sequencing was performed using the Illumina NovaSeq 6000 sequencing platform PE150 (Illumina, USA). The sequencing data output for each sample was not less than 6 G.

### 2.6 RNA-seq data analysis

Clean reads were obtained by removing reads containing adapters and low-quality reads from the raw data. The software hisat2 was used to compare the clean reads with the rice reference genome *Oryza sativa* Japonica Group. Mapped reads are spliced and assembled by using the software StringTie, and functional annotation was carried out through databases such as GO (Gene Ontology), KEGG (Kyoto Encyclopedia of Genes and Genome), and NR (NCBI non-redundant protein sequences). The R language package edgeR was used for gene differential expression analysis, and the DEGs screening threshold was FDR (false discovery rate)<0.05, log_2_FC (fold change)>1 or<-1. The expression of the same gene between the experimental group and the control group at each time point of different inoculation treatments was analyzed. After the genes with no expression difference between the experimental group and the control group were removed, the genes with different expressions between the two treatments and the control group were merged at each time point. DEGs among different inoculation treatments in this gene set were analyzed, and Venn map and volcano map were drawn. GO function annotation and KEGG enrichment analysis of DEGs were carried out by using the Shengxin analysis platform set up by Anhui Microanaly Genetech Co., Ltd. (Hefei, China). The software KOBAS is used to count the abundance of DEGs in the KEGG pathway and analyze the genes and fluxes related to plant disease resistance.

### 2.7 qRT-PCR verification

The genes involved in key pathways from RNA-seq were randomly selected and verified by qRT-PCR. Specific primers of candidate genes were designed by NCBI Primer BLAST (**Table 2**). The reverse transcription experiment was performed using VAHTS HiScript III RT Super Mix for qPCR (R323; Vazyme Biotech Co., Ltd; China). The rice gene *actin* was used as an internal control reference gene, and three technical replicates were set for each gene in each sample. ROCHE Light Cycler 480 SYBR Green I Master (Roche Diagnostics, Germany) reagent was used to prepare the 10 μl PCR reaction system according to the instructions. Gene expression was detected on ABI Quant Studio 6 Flex Real-Time PCR. The relative expression of the selected genes was calculated according to the $2^{-\text{ΔΔCT}}$ method.

**Table 2:** qRT-PCR primer sequence.

Gene name	Forward primer sequence	Reverse primer sequence
*actin*	GAGTATGATGAGTCGGGTCCAG	ACACCAACAATCCCAAACAGAG
*Os01g0172701*	CGGATGGAGTACTGTATAGACC	CGGATGATTGCTGTGGATTATC
*Os02g0110101*	CTGGAACCAGAAGTAGTCGAG	TACCACACGTAGCTGATAAGTC
*Os07g0677500*	ATGGTGAACATGGGGAACATC	CGTCTTATACGTGCATAACTGC
*Os12g0559200*	AAAGGCTCCTTCATATACGAGG	GGTTGTAGTCGATCCAAGAGTT
*Os10g0575100*	CCATCCTAACACAGATCTTT	ATACTCTCTACTCTCCCCAA
*Os02g0597300*	AAATTTGTGGTGGTAATGGAGC	AACATGAATTCACCATTAGCCG
*Os02g0598400*	CTTTGATTTCAAGGGTTGCTCA	CACACTTGAAGAATGACCTTGG

## 3 Result

### 3.1 Introgression segment and background detection of the near-isogenic lines

**Figure 1:**
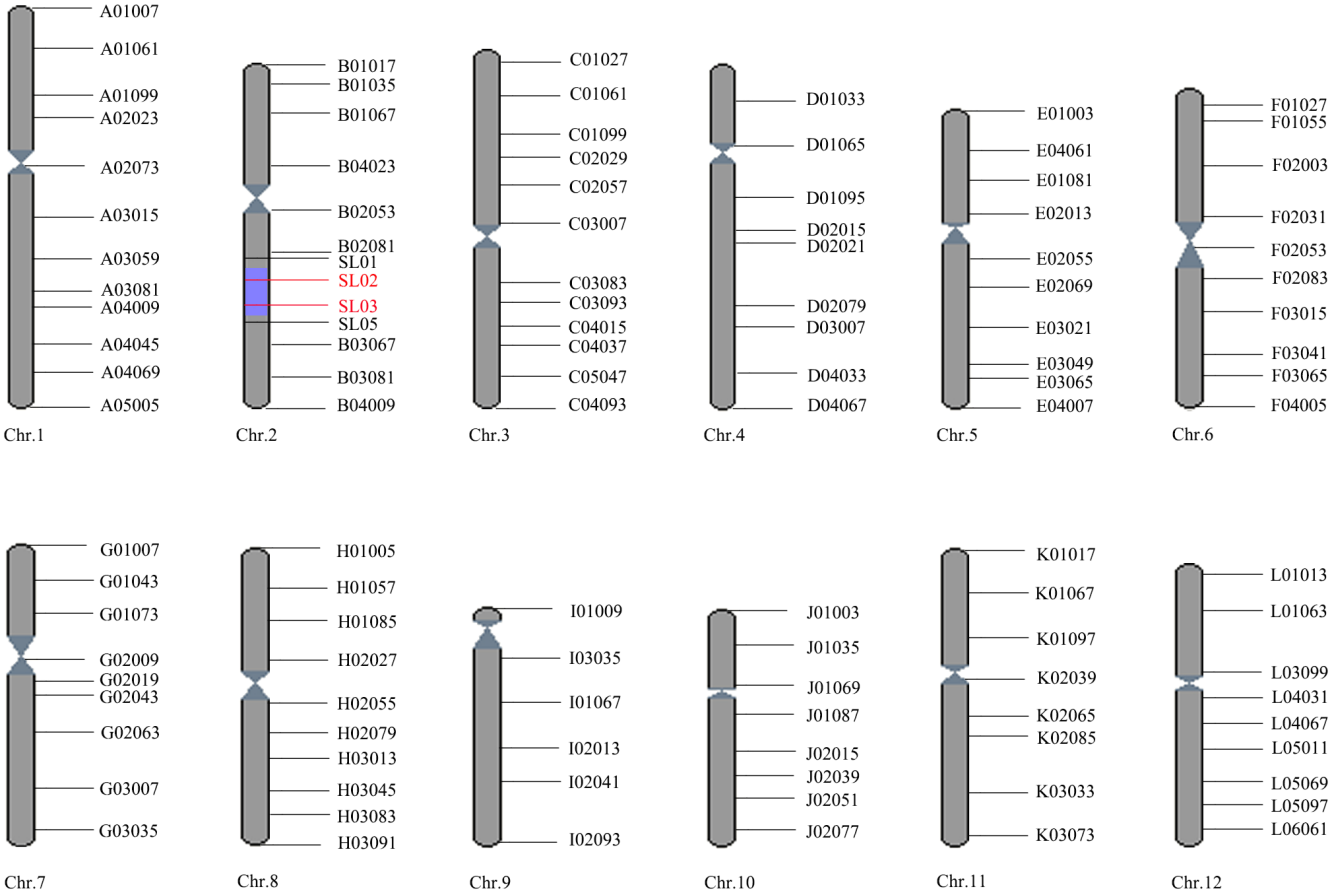
The graphical genotypes of resistant near-isogenic lines (R). The red line indicates markers with the genotype of DY19, while the black line represents markers with the genotype of 9311.The gray cylindrical shows genotype of 9311, and the purple part shows the genotype of DY19 (the segment containing *bls2*).

The BLS resistant and susceptible NILs were constructed by one hybridization, four backcrosses, and two selfings. All plants in the resistant line were uniformly resistant to BLS and having DY19 genotype at molecular marker SL03, while all plants in the susceptible line were consistently susceptible to BLS and exhibiting 9311 genotype at SL03. A total of 116 markers with polymorphism between the donor and recipient parents were used to detect the genotype of the resistant NIL, and the graphical genotype was drawn (**Figure 1**). As shown in **Figure 1**, the genetic background of the resistant NIL was restored to the genotype of the recipient parent 9311 except for the segment containing SL03 on chromosome 2. BLS resistance gene *bls2* should be located on this introgression segment delimited by SL01 and SL05 (spanning∼5.4 M). In addition, we found that the resistant and susceptible near-isogenic lines share the same genetic background, except for this 5.4 M region between SL01 and SL05 on chromosome 2.

### 3.2 Lesion identification of resistant and susceptible near-isogenic lines

**Figure 2:**
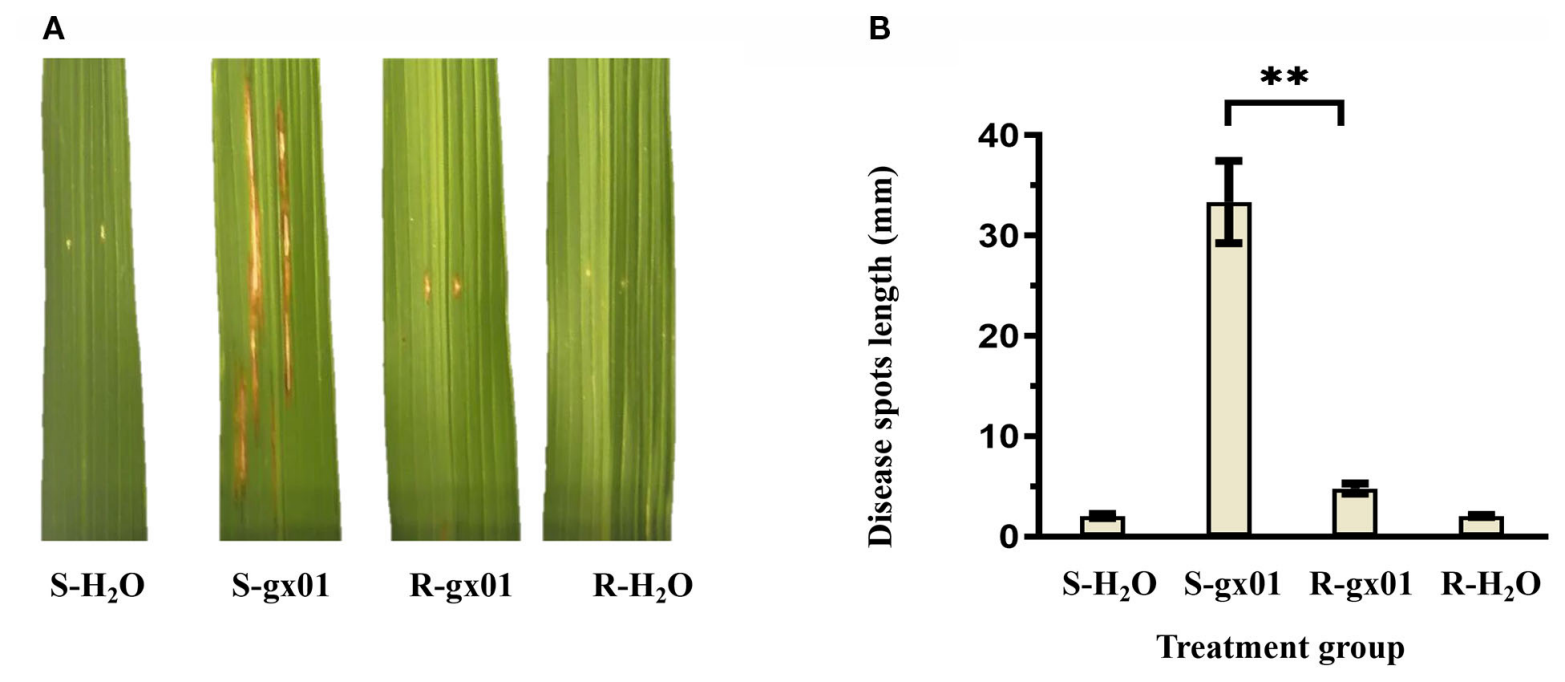
Investigation results of lesions in the experimental and control group of R and S. **(A)** The lesion phenotypes of R and S. **(B)** The lesion lengths of R and S. S-H_2_O and R-H_2_O represent the control group of S and R, respectively. S-gx01 and R-gx01 represent the experimental group of S and R, respectively. The correlation coefficients (Mean, SD and number) were S-H_2_O (2.05mm, 0.22, 9), S-gx01 (33.35mm, 4.08, 9), R-gx01 (4.78mm, 0.50, 9), R-H_2_O (2.05mm, 0.11, 9). Student’s t-test was employed, **Significance at *p*< 0.01.

Rice resistant and susceptible NILs (abbreviated as R and S) were inoculated with *Xoc* strain gx01 (experimental group) and sterile water (control group), respectively. Identification of lesions showed that in the control group of both materials, there were no signs of disease. In the experimental group of R, the average length of lesions was 4.78 mm, showing high resistance to BLS. In the experimental group of S, the average length of lesions was 33.35 mm, showing high susceptibility to BLS (**Figure 2**).

### 3.3 RNA Sequencing Reads

Three biological replicates were designed for each rice materials (R *vs* S), each treatment (*Xoc*-inoculated *vs* sterile water-inoculated), and each time points (12 hpi, 24 hpi,48 hpi). There were 36 RNA samples sequenced on the Illumina platform. The sequencing results meet the analysis requirements: each sample has more than 6G of data, and the quality Q20 of sequencing data is more than 97%. Among the clean reads obtained through data filtering, the number of reads uniquely located in the reference genome was higher than 85%. These unique reads located in the reference genome were used for subsequent gene expression analysis (**Supplementary Table S1**).

### 3.4 Identification and analysis of DEGs

**Figure 3:**
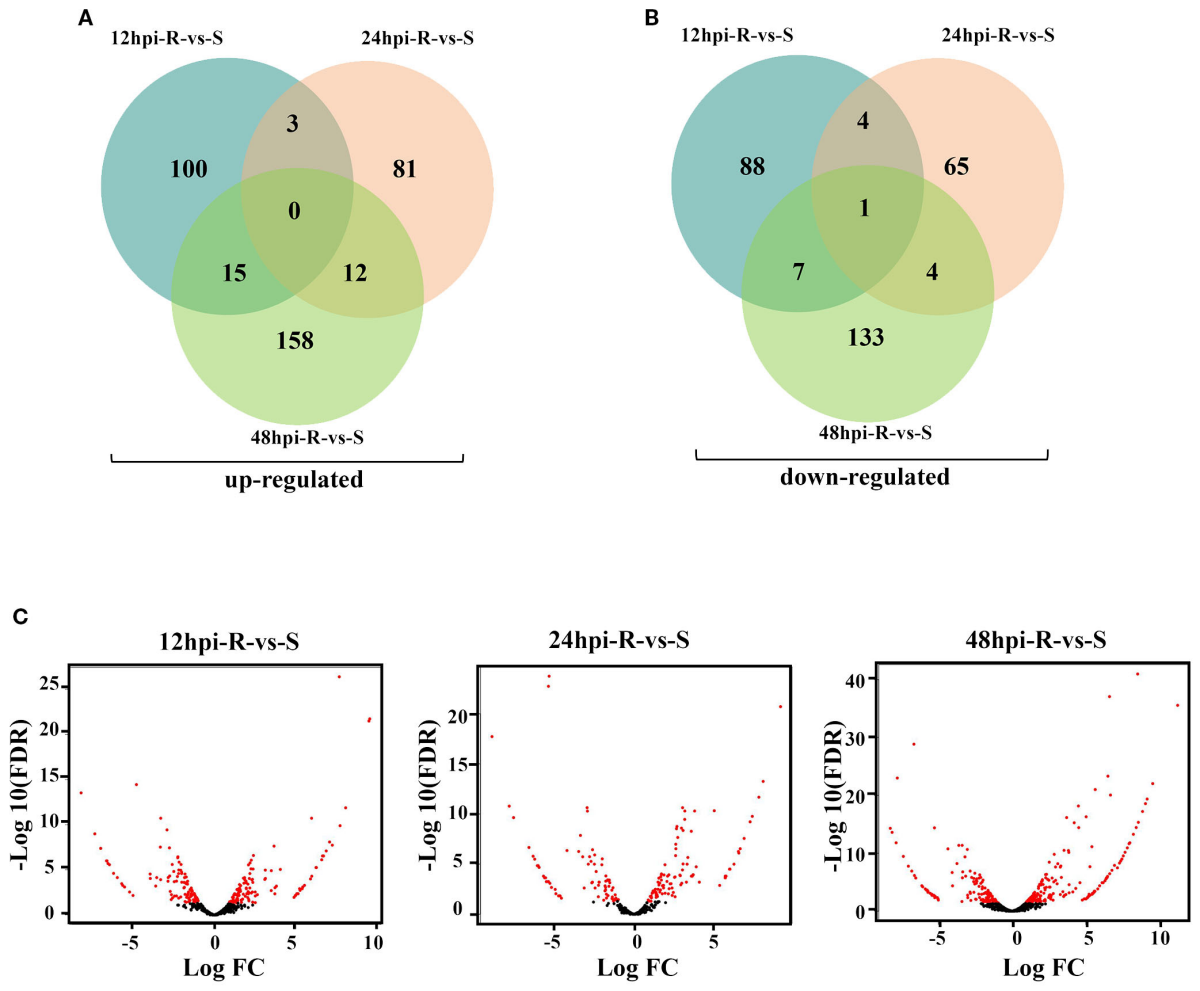
DEGs distribution at different time points in R and S. **(A)** Venn diagram of R-vs-S upregulated genes distribution at different time. **(B)** Venn diagram of R-vs-S down-regulated genes distribution at different time. **(C)** R-vs-S DEGs volcano map at different time.

After removing genes with no expression difference between the experimental group and the control group at each time point of each material, and excluding genes unrelated to BLS resistance response, the DEGs at different time points between R and S were statistically analyzed to reveal the expression pattern of resistance-related genes. A total of 639 DEGs (**Supplementary Table S2**) were identified between R and S at three time points. Namely, 218 DEGs (118 up and 100 down-regulated), 170 DEGs (96 up and 74 down-regulated) and 329 DEGs (185 up and 144 down-regulated) were identified at 12 hpi, 24 hpi and 48 hpi, respectively in R *vs* S. Notably, the number of DEGs was highest at 48 hpi. Furthermore, venn diagrams reveal the number of overlapping DEGs at different time points (**Figure 3**).

### 3.5 DEGs resistance-related gene distribution

DEGs were annotated and those related to plant resistance were screened. Resistance-related genes were divided into five categories (**Supplementary Table S3**): 16 transcription factor related genes (MYB, MYC2, WRKY, ERF, bHLH, etc.), 14 protein kinase-related genes (WAK, CRK, LecRK), 87 resistance protein-related genes (NBS-LRR, LRR, sugar transporter, disease resistance, pathogen-related, calmodulin-like, ubiquitin family, Wax synthesis, sulphate transporter, cytochrome P450, PP2C, glucosyltransferase, cellulose synthase, terpene synthase, flavonol synthase, etc.), 9 hormone-related genes (GAs, IAA, JA, ET, BR) and 4 other genes (CoA). Among these genes related to plant resistance, eight genes were located in the 5.4 M range between SL01 and SL05, and the differential expression was significant (**Table 3**), which were *Os02g0540700* (*OsP3IP1*), *Os02g0569400* (*CYP76M8*), *Os02g0569900* (*CYP76M7*), *Os02g0570700* (*CYP71Z7*), *Os02g0571900* (*CYP76M6*), *Os02g0597300*, *Os02g0599700* (*OsPP2C19*) and *Os02g0621300* (*OsCER1*) respectively. CYP76M8 is a multifunctional/promiscuous hydroxylase, with CYP76M5 and CYP76M7 seeming to provide only redundant activity, while CYP76M6 seems to provide both redundant and novel activity, relative to CYP76M8 (Wang et al., 2012). CYP71Z7 is a C2 oxidase, and Ent-isokaurene C2-hydroxylase performs the initial step in the conversion of ent-isokaurene to the antibacterial oryzalides in rice (Wu et al., 2011). In addition, two identified *Xoc*-TALE target genes showed differential expression: the sulfate transporter gene *Os01g0719300* (*OsSULTR3; 6*) was up-regulated at 24 hpi and down-regulated at 48 hpi; flavanone 3-hydroxylase gene *Os04g0581000* (*OsF3H04g; OsSAH2*) was down-regulated at 12 hpi. Both were known as susceptibility genes for BLS and their downregulated expression may contribute to enhanced disease resistance.

**Table 3:** Gene annotation and differential expression information of DEGs located in the 5.4 M range of SL01 and SL05.

Gene name	R vs S 12h log_2_FC(*P*)	R vs S 24h log_2_FC(*P*)	R vs S 48h log_2_FC(*P*)	Gene annotation
*Os02g0540700*	–	-1.8(1.30E-04)	–	U-box domain-containing protein, Ubiquitin protein
*Os02g0569400*	–	2.6(2.47E-08)	–	Cytochrome P450
*Os02g0569900*	–	2.7(1.55E-09)	–	ent-cassadiene C11-alpha-hydroxylase 1-like, Cytochrome P450
*Os02g0570700*	-2.2(2.45E-06)	2.7(1.73E-10)	–	Cytochrome P450
*Os02g0571900*	–	2.1(1.41E-04)	–	Cytochrome P450
*Os02g0597300*	–	–	6.7 (9.49E-23)	disease resistance protein RPP13, NBS-LRR disease resistance protein (NBS-LRR)
*Os02g0599700*	3.7(6.48E-10)	–	8.5(1.32E-44)	Protein phosphatase 2C-like domain containing protein, Protein Phosphatase 2C (PP2C)
*Os02g0621300*	–	–	11.2(1.03E-38)	very-long-chain aldehyde decarbonylase GL1-4-like, Wax synthesis gene

### 3.6 GO enrichment analysis of DEGs

**Figure 4:**
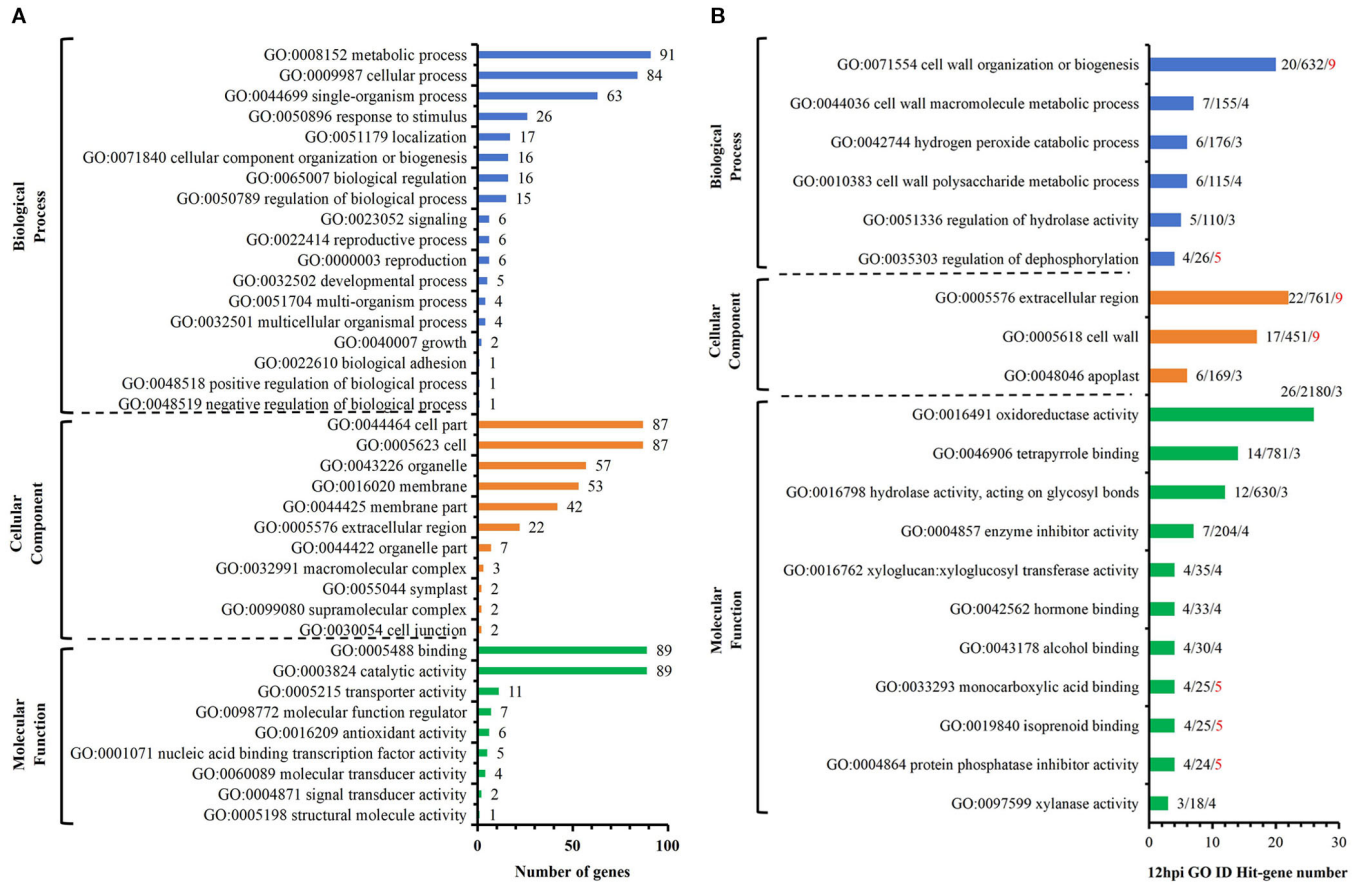
R-VS-S: 12 hpi Go enrichment and significance analysis. **(A)** 12 hpi GO second-level enrichment results. **(B)** 12 hpi GO enrichment significance analysis (*P*<0.01), the tag represents Hit_Genes/background Genes/- log10(*P*). The red font represents the GO term with prominent significance.

**Figure 5:**
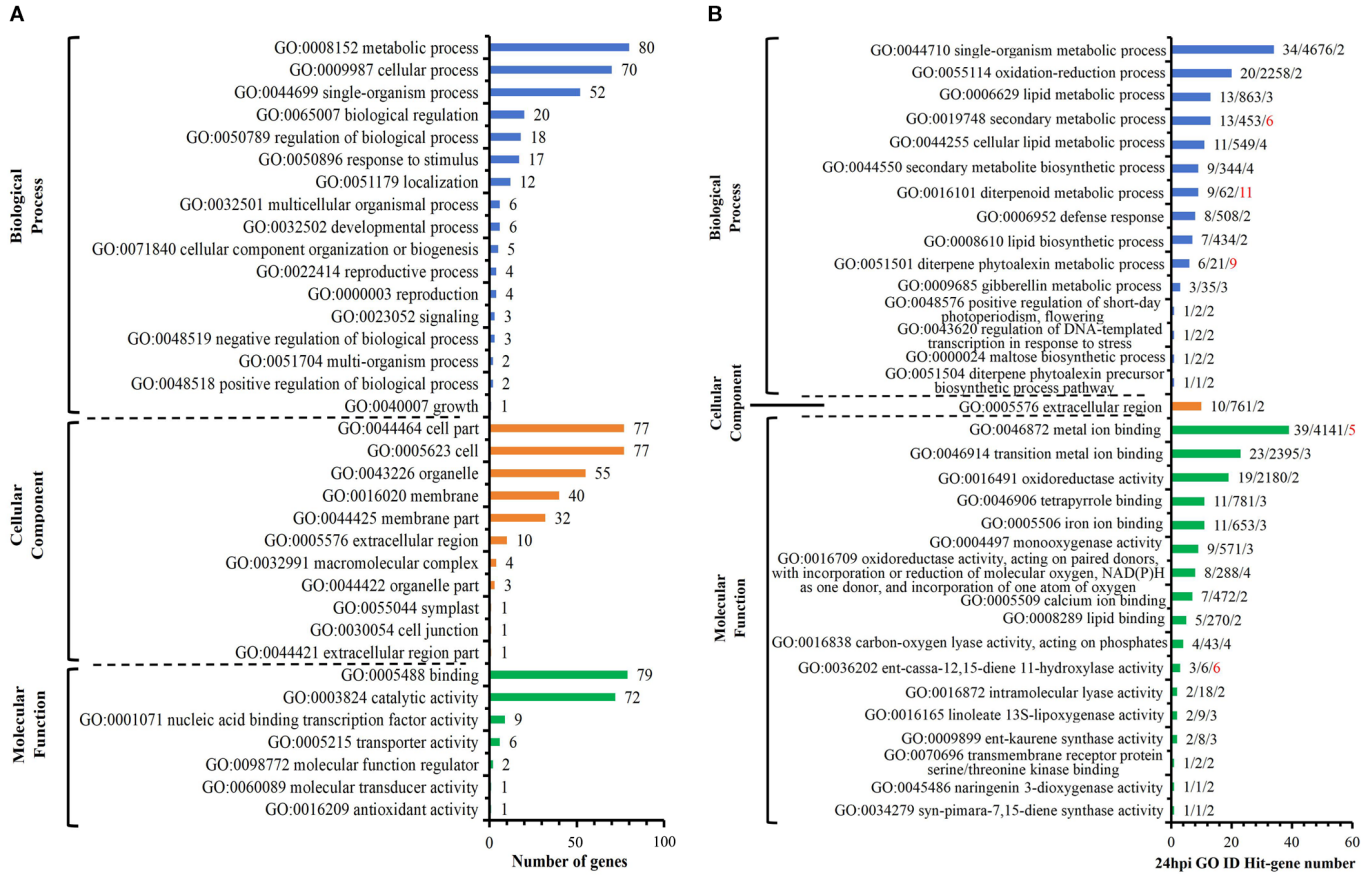
R-VS-S: 24 hpi Go enrichment and significance analysis. **(A)** 24 hpi GO second-level enrichment results. **(B)** 24 hpi GO enrichment significance analysis (*P*<0.01), the tag represents Hit_Genes/background Genes/- log10(*P*). The red font represents the GO term with prominent significance.

**Figure 6:**
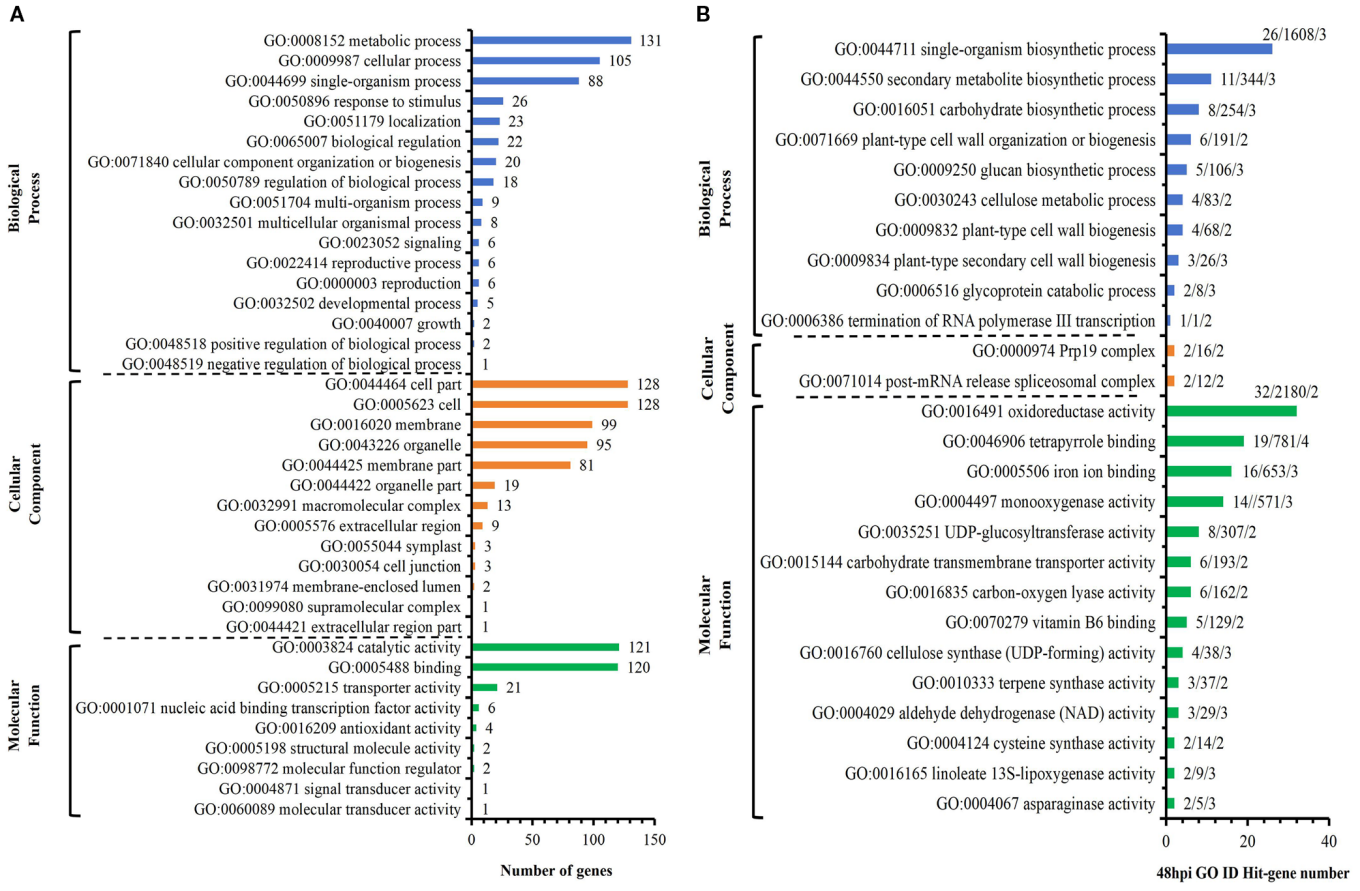
R-VS-S: 48 hpi Go enrichment and significance analysis. **(A)** 48 hpi GO second-level enrichment results. **(B)** 48 hpi GO enrichment significance analysis (*P*<0.01), the tag represents Hit_Genes/background Genes/- log10(*P*).

Among the 218 DEGs identified at 12 hpi, GO annotated 167 genes, including 89 up-regulated genes and 78 down-regulated genes. The second level of GO was enriched to 18 biological process items, 11 cell component items and 9 molecular functions (**Figure 4A**). Significant analysis (P< 0.01) was performed for all enriched levels (**Figure 4B**). Among 6 biological process items, the significance of “GO: 0071554 cell wall organization or biogenesis” and “GO: 0035303 regulation of dephosphorylation” were more prominent; among 3 cell component items, the significance of “GO: 0005576 extracellular region” and “GO: 0005618 cell wall” were more prominent; among 11 molecular functional items, the significance of “GO: 0004864 protein phosphatase inhibitor activity” and “GO: 0019840 isoprenoid binding” and “GO: 0033293 monocarboxylic acid binding” were more prominent. Above analysis indicated that compared with S, R exhibited significant changes in cell wall and extracellular components with active dephosphorylation reactions at 12 hpi. Among the 170 DEGs identified at 24 hpi, GO annotated 133 genes, including 78 up-regulated genes and 55 down-regulated genes. The second level of GO was enriched to 17 biological process items, 11 cell component items and 7 molecular functions (**Figure 5A**). The significance of all enriched levels was analyzed (*P*< 0.01) (**Figure 5B**). Among 15 biological process items, the significance of “GO: 0019748 secondary metabolic process”, “GO: 0016101 diterpenoid metabolic process” and “GO: 0051501 diterpenoid phytoalexin metabolic process” were more prominent; among the 17 molecular functional items, the significance of “GO: 0046872 metal ion binding” and “GO: 0036202 ent-cassa-12,15-diene-11-hydroxylase activity” were more prominent. Above analysis indicated that compared with S, R had more active secondary metabolic process at 24 hpi, especially the diterpenoid phytoalexin metabolic process, which was closely related to disease resistance. Among the 329 DEGs identified at 48 hpi, GO annotated 246 genes, including 134 up-regulated genes and 112 down-regulated genes. The second level of GO was enriched to 17 biological process items, 13 cellular component items and 9 molecular function items (**Figure 6A**). Significant analysis (P< 0.01) was performed for all enriched levels (**Figure 6B**), including 10 biological process items, 2 cellular component items, and 14 molecular function items. Significant GO terms are described as follows: “GO:0044550 secondary metabolite biosynthetic process”, “GO:0009250 glucan biosynthetic process”, “GO:0009832 plant-type cell wall biogenesis”, “GO:0009834 plant-type secondary cell wall biogenesis”, etc. Above analysis indicated that compared with S, R had more active synthesis of the secondary metabolite, glucan, cellulose, cell wall and secondary cell wall at 48 hpi.

### 3.7 KEGG enrichment analysis of DEGs

According to the time point, the identified DEGs were subjected to KEGG metabolic pathway enrichment analysis. KEGG pathways were mainly distributed in five categories: organismal systems, environmental information processing, cellular processes, metabolism and genetic information processing (**Supplementary Table S4**).

Among the 218 DEGs identified at 12 hpi, 68 DEGs were involved in 36 KEGG pathways, including 42 up-regulated genes and 26 down-regulated genes. KEGG enrichment analysis of “ko00592 α-linolenic acid metabolism”, “ko04712 circadian rhythm-plant”, “ko04016 MAPK signaling pathway–plant”, “ko04626 plant-pathogen interaction”, “ko00591 linolenic acid metabolism”, “ko00940 phenylpropanoid biosynthesis”, “ko00908 zeatin biosynthesis” and “ko00350 tyrosine metabolism” were significant (*P*<0.05) (**Supplementary Table S4**). Among them, “ko04712 circadian rhythm-plant” was mainly related to the sampling time and 12 hpi intervals. The specific gene information involved in the significantly enriched KEGG pathways is shown in **Supplementary Table S5**.

Among the 170 DEGs identified at 24 hpi, 46 DEGs were involved in 28 KEGG pathways, including 30 up-regulated genes and 16 down-regulated genes. KEGG enrichment analysis of “ko00904 diterpenoid biosynthesis”, “ko00591 linolenic acid metabolism”, “ko04626 plant-pathogen interaction” and “ko00592 α-linolenic acid metabolism” were significant (*P*<0.05) (**Supplementary Table S4**). By analyzing the specific gene information involved in the related KEGG pathway (**Supplementary Table S6**), it was found that except for the downregulation of calcium-dependent protein kinase (CPK) related gene *Os05g0585601* and calmodulin (CALM) related gene *Os01g0955100* in “ko04626 plant-pathogen interaction” pathway, the other genes were up-regulated. In summary, the biosynthesis of diterpenoids (secondary metabolites associated with resistance) was active in R at 24 hpi, which was consistent with the results of GO enrichment analysis.

Among the 329 DEGs identified at 48 hpi, 103 DEGs were involved in 59 KEGG pathways, including 53 up-regulated genes and 50 down-regulated genes. KEGG enrichment analysis of “ko00902 monoterpene biosynthesis”, “ko00270 cysteine and methionine metabolism”, “ko00592 α-linolenic acid metabolism”, “ko00780 biotin metabolism”, “ko00591 linolenic acid metabolism”, “ko00940 phenylpropanoid biosynthesis” and “ko00950 isoquinoline alkaloid biosynthesis” were significant (*P*<0.05) (**Supplementary Table S4**). And the specific gene information involved in the related KEGG pathways is shown in **Supplementary Table S7**.

### 3.8 Key KEGG pathway analysis

The key KEGG pathways reported to be related to plant disease resistance were analyzed and the relevant DEGs information is listed in **Supplementary Table S8**.

#### 3.8.1 Phenylpropanoid biosynthesis

**Figure 7:**
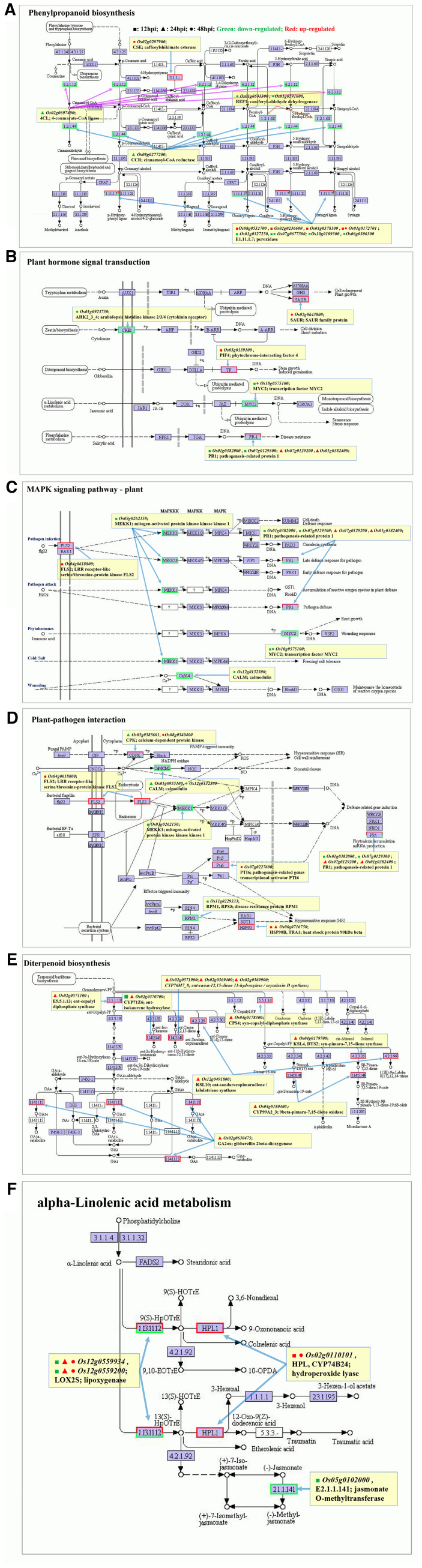
R-vs-S: Important KEGG pathways related to plant disease resistance mechanism. **(A)** Phenylpropanoid biosynthesis pathway; **(B)** Plant hormone signal transduction pathway; **(C)** MAPK signaling pathway-plant pathway; **(D)** Plant-pathogen interaction pathway; **(E)** Diterpenoid biosynthesis pathway; **(F)** α-Linolenic acid metabolism pathway. ■: 12 hpi; ▲: 24 hpi; ●: 48 hpi; Green: down-regulated; Red: up-regulated.

The phenylpropanoid biosynthesis pathway is one of the important secondary metabolic pathways in the plant, and plays important roles in plant defense. There are several enzyme families involved in lignin biosynthesis, including peroxidase, 4-coumarate-CoA ligase (4CL), cinnamoyl-CoA reductase (CCR) and caffeoyl shikimate esterase (CSE) (Dixon et al., 2002). In the “phenylpropanoid biosynthesis” pathway (**Figure 7A**), peroxidase-related genes *Os08g0532700*, *Os02g0236600*, *Os01g0378100* and *Os01g0172701* were up-regulated at 12 hpi, while *Os07g0677500*, *Os10g0109300*, *Os06g0306300* were down-regulated at 48 hpi. In addition, 4CL-related gene *Os02g0697400* and CCR-related gene *Os08g0277200* were down-regulated at 24 hpi, CES-related gene *Os01g0591300* was upregulated at 48 hpi. REF1-related genes *Os01g0591300* (*OsALDH2C1*) and *Os01g0591000* (*OsALDH2C4*; *aLDH1a*, *OsALDH2-1*) were down-regulated at 48 hp. It was speculated that lignin synthesis may be enhanced at 12 hpi but inhibited at 24–48 hpi.

#### 3.8.2 Plant hormone signal transduction

In the “plant hormone signal transduction” pathway (**Figure 7B**), PIF4-related gene *Os05g0139100* (*APG*; *OsPIL16*) was up-regulated at 12 hpi, which promoted gibberellin-mediated stem growth and induced germination. PR1-related genes *Os01g0382000* (*OsPR1b*) and *Os07g0129300* were down-regulated at 12 hpi, while *Os01g0382400* and *Os07g0129200* were up-regulated at 24 hpi, which were involved in salicylic acid-mediated defense response. The SAUR-related gene *Os02g0643800* was up-regulated at 48 hpi, which promoted auxin-mediated cell enlargement and plant growth. The AHK2_3_4-related gene *Os01g0923750* was down-regulated at 48 hpi, inhibiting cytokinin-mediated cell division and shoot initiation. The transcription factor MYC2-related gene *Os10g0575100* was down-regulated at 12 hpi and 48 hpi, affecting jasmonic acid-mediated monoterpene biosynthesis, indole alkaloid biosynthesis, senescence, and stress responses. This gene was also involved in the “plant MAPK signaling” pathway (**Figure 7C**).

#### 3.8.3 Plant MAPK signaling

In the “plant MAPK signaling” pathway (**Figure 7C**), the FLS2-related gene *Os04g0618800* was up-regulated at 12hpi, facilitating the binding of FLS2 to bacterial flagellin flg22; the MEKK1-related gene *Os03g0262150* was down-regulated at 12 hpi, inhibiting signal amplification through phosphorylation (the first level) to transmit external stimuli into the cell. Pathogenesis protein PR1-related genes *Os01g0382000* (*OsPR1b*) and *Os07g0129300* were down-regulated at 12 hpi, while *Os01g0382400* and *Os07g0129200* were up-regulated at 24 hpi, participating in the defense response induced by bacterial flagellin flg22. It was estimated that 0 ~ 12hpi was the stage of pathogen invasion, while during 12~24 hpi, the plant immune response was triggered to combat the pathogen attack. The CALM-related gene *Os12g0132300* (*OsCML3*) was down-regulated at 48 hpi and involved in the active oxygen homeostasis response mediated by Ca^2+^ at the wound site.

#### 3.8.4 Plant-pathogen interaction

In the “plant-pathogen interaction” pathway (**Figure 7D**), six DEGs involved in PTI immune signaling pathways were identified. the FLS2-related gene *Os04g0618800* (up-regulated at 12 hpi) perceived the bacterial flagellin; the MEKK1-related gene *Os03g0262150* (down-regulated at 12 hpi) participated in the signaling tandem of MAPK cascades; CPK-related genes *Os05g0585601* (down-regulated at 24 hpi) and *Os08g0540400* (*OsCPK21*; *OsCDPK21*) (up-regulated at 48 hpi), CALM-related genes *Os01g0955100* (*OsMSR2*; *OsCML31*) (down-regulated at 24 hpi) and *Os12g0132300* (*OsCML3*) (down-regulated at 48 hpi) participated in the accumulation of ROS or nitric oxide (NO) induced by Ca^2+^, thereby triggering hypersensitivity or cell wall enhancement. Three DEGs involved in ETI immune signaling pathways were identified. The PTI6-related gene *Os07g0227600* (up-regulated at 48 hpi) induced the expression of defense-related genes. The disease resistance protein RPM1-related gene *Os11g0229333* was down-regulated at 12 hpi, suppressing the hypersensitive reaction induced by bacterial secretions. The heat shock protein HSP90B related gene *Os06g0716750* was significantly up-regulated at 12 hpi and 24 hpi, promoting the hypersensitivity reaction caused by bacterial secretions.

#### 3.8.5 Diterpene biosynthesis

In the “diterpene biosynthesis” pathway (**Figure 7E**), among all DEGs, Only CYP71Z-related gene *Os02g0570700* were down-regulated at 12 hpi, while all others were up-regulated at 24 hpi, mostly on chromosome 2 and chromosome 4. On chromosome 2, the CPS-related gene *Os02g0571100* (*OsCPS2*; *OsCyc2*), CYP76M-related genes *Os02g0571900* (*CYP76M6*), *Os02g0569400* (*CYP76M8*), *Os02g0569900* (*CYP76M7*) and CYP71Z-related gene *Os02g0570700* were up-regulated at 24hpi, which jointly promoted the synthesis of diterpenoid plant antitoxins such as phytocassanes. The GA2ox-related gene *Os02g0630475* was up-regulated to promote the synthesis of gibberellin. On chromosome 4, the CPS4-related gene *Os04g0178300* (*OsCPS4*; *OsCyc1*), KSL4-related gene *Os04g0179700* (*OsDTS2*; *OsKSL4*; *OsKS4*), and CYP99A-related gene *Os04g0180400* (*CYP99A2*) were up-regulated, which promoted the synthesis of diterpenoid plant antitoxins such as momilactone A. In addition, the KSL-related gene *Os12g0491800* (*OsKSL6*;*OsKSL10*; *OsTPS21*) on chromosome 12 was up-regulated at 24 hpi, which promoted the synthesis of diterpenoid plant antitoxins such as oryzalexins A-F.

#### 3.8.6 α-linolenic acid metabolic

In the “α-linolenic acid metabolic” pathway (**Figure 7F**), the lipoxygenase LOX2S-related genes *Os12g0559200*(*OsLOX11*; *OsRCI-1*) and *Os12g0559934* were down-regulated at 12 hpi and up-regulated at 24 hpi and 48 hpi. Jasmonate O-methyltransferase-related gene *Os05g0102000* (*OsJMT1*)was down-regulated at 12 hpi, and the HPL (hydroperoxide lyase) related gene *Os02g0110101* was up-regulated at 12 hpi and 48 hpi, which involved in the biosynthesis of jasmonate, traumatic acid and so on. It was speculated that the synthesis of these substances may be inhibited at 12 hpi and gradually increased from 24 hpi to 48 hpi.

### 3.9 qRT-PCR confirms gene expression profiles

**Figure 8:**
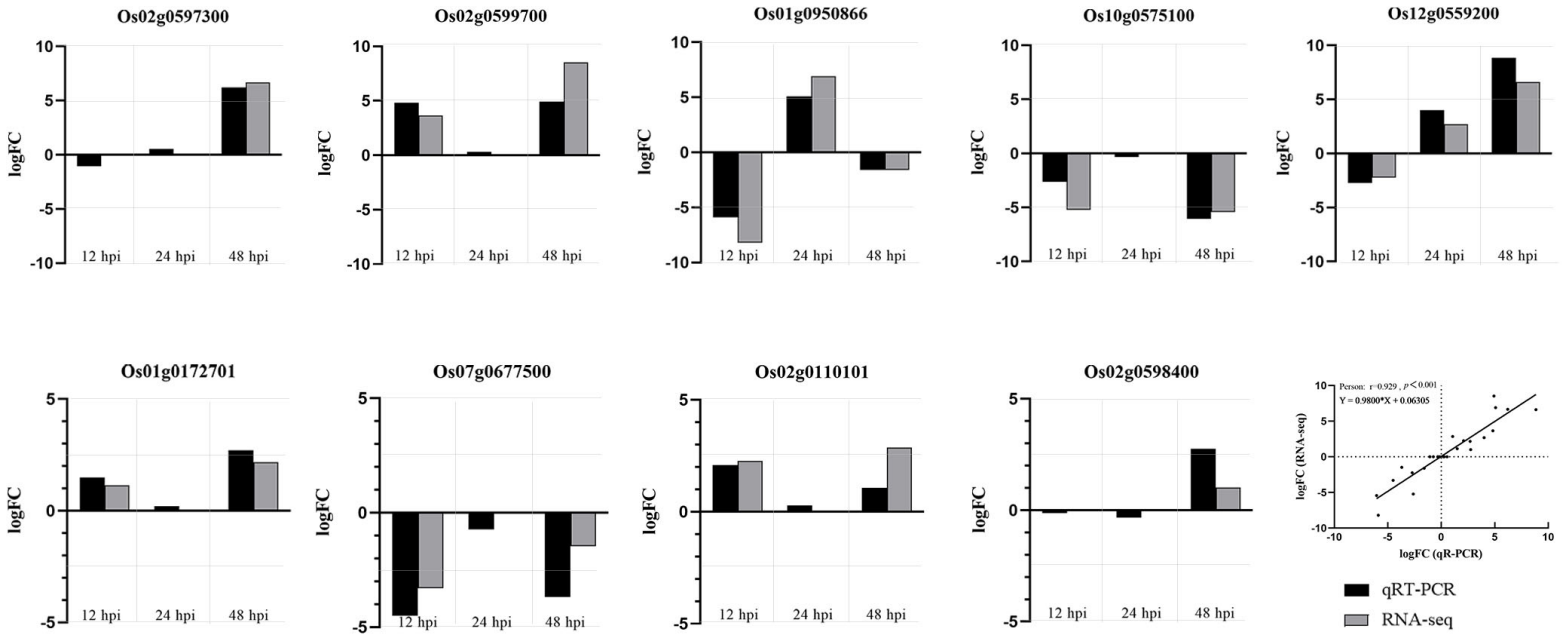
R-VS-S qRT-PCR validation results and RNA-seq sequencing results. The expression trend of qRT-PCR results was consistent with RNA-seq sequencing results.

According to the results of RNA-seq analysis, 9 genes (*P*< 0.01) were randomly selected for qRT-PCR verification. They were the NBS-LRR resistance protein-related gene *Os02g0597300*; a protein phosphatase 2C-related gene *Os02g0599700*; a expressed protein gene *Os02g0598400*; a proline synthetase-related gene *Os01g0950866*; peroxidase-related genes *Os01g0172701* and *Os07g0677500*, which were involved in lignin synthesis through “phenylalanine synthesis” pathway; hydroperoxide lyase related gene *Os02g0110101* and lipoxygenase related gene *Os12g0559200* in “α-linolenic acid metabolic” pathway; transcription factor MYC2 related gene *Os10g0575100*, which was involved in defense response mediated by jasmonic acid in “plant hormone signal transduction” and “plant MAPK signal pathway”. The expression trend of qRT-PCR results was consistent with RNA-seq sequencing results, which indicates that RNA-seq sequencing results are reliable (**Figure 8**).

## 4 Discussion

### 4.1 PTI and ETI trigger defense responses

*Xoc* is a pathogenic variant of the *Xanthomonas* family in the genus *Xanthomonas*. It uses its flagella to move and invades rice mainly through leaf wounds or stomata. The RLK Flagellin-sensitive 2 (FLS2) of Arabidopsis thaliana can recognize the flagellin fragment flg22 (Gómez-Gómez and Boller, 2000). the flg22 peptide directly binds to FLS2 and triggers the recruitment of the related receptor kinase BAK1 (Chinchilla et al., 2007). It is indicated that flg22 can induce the formation of a complex between FLS2 and BAK1 to initiate PTI reaction (Sun et al., 2013). In our study, six DEGs were identified that were related to the PTI pathway. When the highly pathogenic strain gx01 infected rice, compared with S, R up-regulates the protein kinase FLS2-related genes to enhance their ability to recognize and bind bacterial flagellin flg22 at 12 hpi. Phosphorylated BAK1 dissociates from the complex and activates downstream multiple immune responses, such as promoting ROS burst (Kadota et al., 2014). The activation of FLS2 downstream signaling triggers the influx of calcium ions from the extracellular to the intracellular: on the one hand, it activates the anion channels on the plasma membrane, promoting the exudation of alkaline substances for antimicrobial activity; on the other hand, it activates CPKs to regulate ROS burst. The MAPK cascade pathway is also involved in FLS2-mediated signal transduction (Bethke et al., 2012). We found that in the “plant-pathogen interaction” and “MAPK signaling” pathway, MEKK1-related gene was enriched at 12 hpi, Ca^2 +^ mediated CPKs-related genes and CALMs-related genes were enriched at 24 hpi and 48 hpi (**Figures 7C, D**). These DEGs may involve in PTI immune signaling pathways to provide BLS resistance.

To overcome virulence factors, plants have evolved disease resistance (R) genes to recognize effectors from pathogens and initiate ETI. RPM1 belongs to the NBS-LRR class of R gene proteins that acts as a signalling adaptor for the pathogen avirulence gene (*avrRpm1*) product, leading to the oxidative burst and hypersensitive cell death (Grant et al., 2000). In our study, Three DEGs were enriched in ETI pathway: RPM1-related gene *Os11g0229333* was down-regulated at 12 hpi. The heat shock protein HSP90B gene *Os06g0716750* was up-regulated at 12 hpi and 24 hpi, which may cause HR to against the pathogen invasion. Pti6 *Os07g0227600* was up-regulated at 48 hpi, which functioned as transcription factors for regulating ETI process. Taken together, *bls2* was proposed to regulate both PTI- and ETI-related genes, initiating a series of defense responses. Compared to S, most genes involved in PTI were up-regulated at 12 hpi but down-regulated at 24 and 48 hpi in R; whereas most ETI-related genes showed down-regulation at 12 hpi but up-regulation at 24 and 48 hpi in R. It suggests that PTI primarily occurs at 12 hpi while ETI mainly functions at 24–48 hpi in R.

In our study, two TALE target genes exhibited differential expression between R and S. For *Xoc*, very few TALE virulence target genes have been reported (Bi et al., 2024). The sulfate transporter gene *Os01g0719300*(*OsSULTR3; 6*) targeted by Tal2g is a major susceptibility gene for BLS (Cernadas et al., 2014). Editing EBE in the *OsSULTR3;6* promoter region enhanced rice resistance to BLS (Xu et al., 2021). In this study, it was up-regulated at 24 hpi and down-regulated at 48 hpi, indicating a gradual decrease of its expression level in R from 24 to 48 hpi, thereby enhancing resistance to BLS. The flavanone 3-hydroxylase gene *OsF3H04g* is another BLS susceptibility gene targeted by Tal2c. Overexpressing *OsF3H04g* caused higher susceptibility and less SA production compared to wild-type plants (Wu et al., 2021). We found that *OsF3H04g* was down-regulated at 12 hpi. It was consistent with the above report.

### 4.2 Jasmonate and salicylate pathways synergistically regulate defense gene expression

α-linolenic acid metabolites are involved in the synthesis of the plant hormone jasmonic acid. Jasmonic acid not only induces physiological changes in plants to form defensive structures but also induces the expression of downstream defensive genes in signaling pathways, thereby enhancing plant resistance to pathogens. In our study, the genes enriched in the “α-linolenic acid metabolic” pathway were up-regulated at 24 hpi and 48 hpi. indicating that the synthesis of jasmonate and traumatic may be gradually increased from 24 hpi to 48 hpi. In the “plant hormone signal transduction” pathway, the transcription factor MYC2-related gene *Os10g0575100* was down-regulated at 12 hpi and 48 hpi. MYC2 is an important regulator in the jasmonic acid signaling pathway. AtMYC2 can reduce the defense of Arabidopsis thaliana against bacterial pathogens (Song et al., 2017). Giri et al. (2017) generated multiple transgenic rice lines for over-expression and RNAi-mediated suppression of *OsMYC2*, then found that OsMYC2 is a negative regulator of rice *Xoo* resistance. In this study, *Os10g0575100* was consistently highly down-regulated, indicating that the inhibition of the jasmonic acid pathway was broken and the genes involved in jasmonic acid response were activated. Moreover, PR1-related genes *Os01g0382400* and *Os07g0129200* were up-regulated at 24 hpi, which were involved in the salicylic acid-mediated defense response. The above results suggested that R activated jasmonic acid and salicylic acid-dependent signal transduction pathways to trigger defense responses against *Xoc* invasion at 24 hpi.

### 4.3 Diterpenoid phytoalexins provide chemical defense against pathogen invasion

Almost all of the identified natural phytoalexins in rice belong to diterpenoids (Schmelz et al., 2014). Among them, the P450s involved in plant terpenoid biosynthesis are mainly distributed in the three families of CYP71, CYP85 and CYP72, among which the CYP71 family is involved in most plant secondary metabolism (Hamberger and Bak, 2013). The infection of *M.oryzae* can strongly induce the accumulation of these diterpenoid antitoxins (Hasegawa et al., 2010). In our study, at 24 hpi, the diterpene plant antitoxin metabolic reaction was more active, and a large number of up-regulated genes were enriched in the diterpene biosynthesis pathway. These genes are mainly involved in diterpene skeleton synthesis and sitosterol synthesis pathways, indicating that R resist *Xoc* infection through large-scale synthesis and accumulation of diterpene plant antitoxins. It is worth noting that in the diterpenoid biosynthetic pathway, a large number of differentially enriched genes are mainly concentrated on chromosome 2 and chromosome 4, which is highly consistent with the previous studies that rice carries two such gene clusters for the production of antimicrobial diterpenoid phytoalexins (Shimura et al., 2007; Wang et al., 2012).

### 4.4 Resistance-related genes on introgression segment of the resistant NIL

Combined with background detection of R, eight DEGs, Cytochrome P450 gene *Os02g0569400* (*CYP76M8*), *Os02g0569900* (*CYP76M7*), *Os02g0570700* (*CYP71Z7*) and *Os02g0571900* (*CYP76M6*), P3-inducible U-box type E3 ubiquitin ligase gene *Os02g0540700* (*OsP3IP1*), Protein phosphatase 2C-related gene *Os02g0599700 (OsPP2C19*), NBS-LRR gene *Os02g0597300* and Wax synthesis gene *Os02g0621300* (*OsCER1*) were located in the 5.4 M range of SL01 and SL05. P450s are the largest family of enzymes in plant metabolism. P450s in the same family or subfamily can catalyze the continuous steps of the same pathway and can also catalyze similar reactions of different substrates. *CYP71Z18* overexpression confers elevated blast resistance in transgenic rice (Shen et al., 2019). Cyt02 encodes cytochrome P450 monooxygenase, increasing rice (*Oryza sativa L*.) resistance to sheath blight (Zheng et al., 2025). Knockdown of CYP76M7 and CYP76M8 simultaneously inhibited elicitor-induced phytocassanes production (Wang et al., 2012). Deletion of diterpenoid biosynthetic genes *CYP76M7* and *CYP76M8* induces cell death and enhances bacterial blight resistance in 9311 (Jiang et al., 2022). In our study, *CYP76M8, CYP76M7 and CYP76M6 were* up-regulated at 24 hpi and *CYP71Z7 was* down-regulated at 12 hpi and up-regulated at 24hpi, indicating that at 24 hpi, large-scale synthesis and accumulation of diterpenoid phytoalexins were occurring in R cells to resist *Xoc* infection. OsP3IP1 has E3 ligase activity, interacts with OsNRPD1a and OsNRPD1b to mediate their ubiquitination, and degrades them through the ubiquitin-proteasome system, thereby negatively regulating rice resistance to rice grassy stunt virus (RGSV) (Zhang et al., 2020). In our study, *OsP3IP1* down-regulated at 24 hpi. It could be inferred that the gene may negatively regulate rice resistance to BLS. Protein phosphatase 2C (PP2C), a group of Ser/Thr-specific phosphatases dephosphorylating target proteins, have implications in plant immunity (Bhaskara et al., 2019). *OsPP2C41*, which plays positive roles in rice blast resistance and chitin-triggered immune responses (Wang et al., 2024). In our study, *OsPP2C19* was up-regulated at 12 hpi and 48 hpi, suggesting that the gene affect resistance to BLS. The NBS-LRR domain has become an important component of plant defense against microbial invasion during the long-term co-evolution between plants and pathogens. The cloned BLS-resistance genes *Xo1* and *Rxo1* both contain the NBS-LRR domain (Read et al., 2020; Zhao et al., 2005). In our study, NBS-LRR gene *Os02g0597300* was up-regulated at 48 hpi, which is likely to be a potential component of *bls2* and requires further research. The outermost surfaces of plants are covered with an epicuticular wax layer that provides a primary waterproof barrier and protection against different environmental stresses. *OsCER1* is a key gene in wax biosynthesis and plays an indispensable role in cold tolerance during the booting stage of rice (Hou et al., 2024). In our study, *OsCER1 was* up-regulated at 48hpi and secondary cell wall synthesis and cellulose synthesis in R were more active at 48 hpi. We speculate that these eight genes may be related to the resistance to BLS.

## 5 Conclusions

The molecular interaction mechanism of R and S in response to infection by *Xoc* strain gx01 was analyzed by transcriptome sequencing. Upon inoculation, compared with S, R up-regulated the expression of FLS2-related genes involved in PTI, modulating MAPK cascade pathway MEKK1-related genes at 12 hpi. At 24 hpi, a large number of diterpene phytoalexins were synthesized in R cells, activate jasmonic acid and salicylic acid-dependent signal transduction pathways, and ETI-related HSP90B gene was up-regulated. At 48 hpi, the defense reaction was carried out in R cells by strengthening the cell wall, enhancing the synthesis of jasmonic acid, synthesizing monoterpene compounds and isoquinoline alkaloids. Taken together, *bls2* was proposed to regulate both PTI- and ETI-related genes through multi-level defense system, including plant hormone-mediated regulation, antimicrobial phytoalexin biosynthesis, and structural barrier reinforcement. It provides new data resources and theoretical basis for exploring the infection mechanism of *Xoc* strain gx01 and the resistance mechanism of resistance gene *bls2*.

## Data Availability

The datasets presented in this study can be found in online repositories. The names of the repository/repositories and accession numbers can be found in the article/**Supplementary Material**.
